# Voices in Motion: Results From a Community Choir Intervention to Promote Living Well With Dementia

**DOI:** 10.1093/geroni/igaa057.3033

**Published:** 2020-12-16

**Authors:** Debra Sheets, Stuart MacDonald, Theresa Allison

## Abstract

Dementia is recognized as a global public health priority because of the significant impact it has on individuals, families and society. The numbers of people living with dementia worldwide are currently estimated at 35.6 million; this will double by 2030 and more than triple by 2050. Given the lack of a medical cure for dementia, lifestyle interventions to complement existing treatment are urgently needed to support living well with this disease. Engagement in the arts is a novel intervention which is relatively low cost and engaging. This study examines the effect of participation in an intergenerational choir on psychosocial and cognitive function for persons with dementia (PwD). Participants (n = 32), in partnership with their family caregivers and local high school students, sang in a professionally conducted choir for as many as three seasons (~ 12 weeks long) spanning up to 18 months of follow-up. Assessments of psychosocial, physiological, and cognitive function were completed every four to six weeks as part of an intensive repeated measures design. Taken as a whole, the symposium papers indicate that this novel lifestyle intervention offers an effective non- pharmacological alternative approach for older adults with dementia. Choir participation has important and significant impacts on psycho-social well-being and quality of life. Discussion focuses on policy implications and the need for community-based programs that reflect a social model for dementia and support living well by through engaging and meaningful activities

